# Is RDW a clinically relevant prognostic factor for newly diagnosed multiple myeloma? A systematic review and meta-analysis

**DOI:** 10.1186/s12885-022-09902-9

**Published:** 2022-07-19

**Authors:** Xiaomin Chen, Jiayue Liu, Jialin Duan, Hao Xiong, Yang Liu, Xinwen Zhang, Chunlan Huang

**Affiliations:** Stem Cell Laboratory, Affiliated Hospital of Southwest Medical University, Luzhou City, Sichuan Province China

**Keywords:** Multiple myeloma (MM), Red cell distribution width (RDW), Prognosis, Meta-analysis

## Abstract

**Background:**

Multiple myeloma (MM) is an incurable plasma cell malignancy. Red cell distribution width (RDW) is a prognostic marker in various diseases, solid tumors, and hematologic neoplasms, but its prognostic significance in MM is controversial. In this study, we aimed to assess the relationship between RDW and the clinical prognosis of MM patients through a meta-analysis.

**Methods:**

Relevant literature were retrieved from PubMed, Embase, and Web of Science databases according to PRISMA guideline. All relevant parameters were extracted and combined for statistical analysis. The effect size was presented as hazard ratio (HR)/odds ratio (OR) and 95% confidence interval (CI). HR/OR > 1 in MM patients with high RDW suggested a worse prognosis. Heterogeneity test evaluation was performed using Cochran's Q test and I2 statistics. A *P*_heterogeneity_ < 0.10 or *I*^2^ > 50% suggested significant heterogeneity. *P* < 0.05 was considered statistically significant. Statistical analysis was performed using Stata 12.0 software.

**Results:**

8 articles involving 9 studies with 1165 patients were included in our meta-analysis. Our results suggested that elevated RDW is significantly associated with poor prognosis in MM (OS: HR = 1.91, 95%CI: 1.48–2.46; PFS: HR = 2.87, 95% CI: 2.02–4.07). A significant correlation was not found between RDW and International Staging System (ISS) staging (ISS III VS ISS I-II: OR:1.53; 95%CI:0.97–2.42).

**Conclusion:**

Our results suggested that RDW is a robust predictor of newly diagnosed MM outcomes.

## Introduction

Multiple myeloma (MM), the second most common hematologic malignancy after non-Hodgkin's lymphoma in adults worldwide [1], is characterized by abnormal accumulation of malignant plasma cells in the bone marrow and excessive production of monoclonal immunoglobulins [2]. Proteasome inhibitors and immunomodulatory drugs, the primary agents currently used in the treatment of multiple myeloma [2], have significantly improved the survival of patients [3]. With the introduction of therapies such as autologous stem cell transplantation (ASCT) and CD38 monoclonal antibodies (e.g., Daratumumab), the overall treatment of MM has gradually achieved good results, with patients achieving deeper depth of remission and longer overall survival [4–6]. However, it remains an incurable disease due to genetic heterogeneity and other reasons [7]. Therefore, there is a pressing need to identify the high-risk patients with poor prognoses so that novel and intensive protocols can be initiated earlier to improve survival.

Currently, The Revised International Staging System (R-ISS) is a powerful prognostic staging system commonly used for MM and an authoritative reference for treatment strategies [8], which is proposed by the International Myeloma Working Group (IMWG) based on International Staging System (ISS), high-risk cytogenetics [t(4;14), t(14;16) and del17p by interphase fluorescent in situ hybridization (iFISH)] and elevated serum lactate dehydrogenase (LDH) [9]. However, patients with the same stage who received similar treatment regimens still had dramatically different clinical outcomes. It suggests that the current staging system is not comprehensive enough in predicting survival outcomes and reflecting the biological heterogeneity of MM patients. In addition, fluorescence in situ hybridization (FISH) assays are very expensive. Therefore, there is significant interest in investigating readily accessible biomarkers to estimate survival outcomes in MM.

Increasing evidence suggests that the systemic inflammatory response may play an essential role in the development of tumors [10]. The prognostic value of inflammatory factors such as C-reactive protein, neutrophil to lymphocyte ratio (NLR), and red blood cell distribution width (RDW) has been assessed in various types of cancer. RDW, which reflects the variability of red blood cell size, has been reported to be associated with a worse prognosis in a variety of cancers, including pancreatic [11], colorectal [12], and breast cancers [13].

Recent studies have shown that RDW is a potential prognostic marker in MM. However, based on their findings, the opinion about the prognostic value of RDW in MM remains inconclusive. It is generally acknowledged that meta-analysis is a powerful statistic tool to overcome the limitation of different sample sizes from individual studies and generate the best estimation. In this study, we aimed to draw the prognostic value of elevated RDW in MM.

## Methods

### Search strategy

We have conducted a comprehensive literature search of articles through the PubMed, Embase, and Web of Science databases with no date or language restrictions. The last search was conducted on March 25, 2022. The main search terms included: (“RDW” OR “red blood cell distribution width” OR “red cell distribution width”) AND (“multiple myeloma” OR “Myeloma”) AND (“prognosis” OR “prognostic” OR “outcome” OR “survival” OR “predict” OR “prediction”). Furthermore, we also checked the relevant articles in the reference list.

### Inclusion and exclusion criteria

The inclusion criteria for this meta-analysis to select study are as follows: (1) studied patients with MM were diagnosed according to the criteria of the International Myeloma Working Group in 2014. The following inclusion criteria were used to select studies for this meta-analysis:(1) The MM patients of studies were identified according to the International Myeloma Working Group 2014 diagnostic criteria. [14]. (2) Association between RDW and overall survival (OS) and progression-free survival (PFS) was reported. (3) RDW was measured by blood-based methods without any formal treatment. The exclusion criteria are as follows: (1) abstracts, letters, meta-analysis, case reports, or reviews. (2) basic studies or animal trials. (3) studies with insufficient data for estimating hazard ratio (HR) and 95% confidence interval (CI). (4) patients suffered from other primary tumors, severe infections, or relapsed MM.

### Data extraction and quality assessment

Two independent authors evaluated and extracted all candidate articles (Xiaomin Chen and Jiayue Liu). Articles that could not be categorized based on title and abstract were retrieved for full-text review. If disagreement occurred, two authors discussed and arrived at a consensus with a third author (Jialin Duan). For each study, the following items were recorded: first author, year of publication, country, sample size and gender, age, follow-ups, cut-off value, treatment strategy, ISS stage, and HRs with 95%Cis. The Newcastle–Ottawa Scale (NOS) was used to assess each of the included studies’ quality by two independent authors (Xiaomin Chen and Jialin Duan) [15]. The NOS consists of three parts: selection (0–4 points), comparability (0–2 points), and outcome assessment (0–3 points). NOS scores of ≥ 7 were assigned as high-quality studies.

### Statistical analysis

HRs and 95%CIs were obtained directly from each of the publications. HR > 1 in MM patients with high RDW suggested a worse prognosis. The relationship between RDW and clinicopathological parameters was evaluated by the combined odds ratio (OR) and its 95%CIs. Cochran’s Q test and Higgins I-squared statistical methods were used to evaluate the heterogeneity of the included studies. A *P*_heterogeneity_ < 0.10 or *I*^2^ > 50% suggested significant heterogeneity [16]. Both the fixed-effects (Mantel–Haenszel method) model and the random effects (DerSimonian–Laird method) model were used to calculate the combined HRs and 95%CIs [17]. The sources of heterogeneity were explored using subgroup analysis. Sensitivity analysis was used to evaluate the stability of the combined results. Publication bias was assessed by Begg’s funnel plot and Egger’s linear regression test [18, 19]. All p-values were bilateral, and *p* < 0.05 was considered statistically significant. All the data were analyzed by STATA12.0 software (STATA, Colleges Station, TX).

## Results

### Study characteristics

**Fig. 1:**
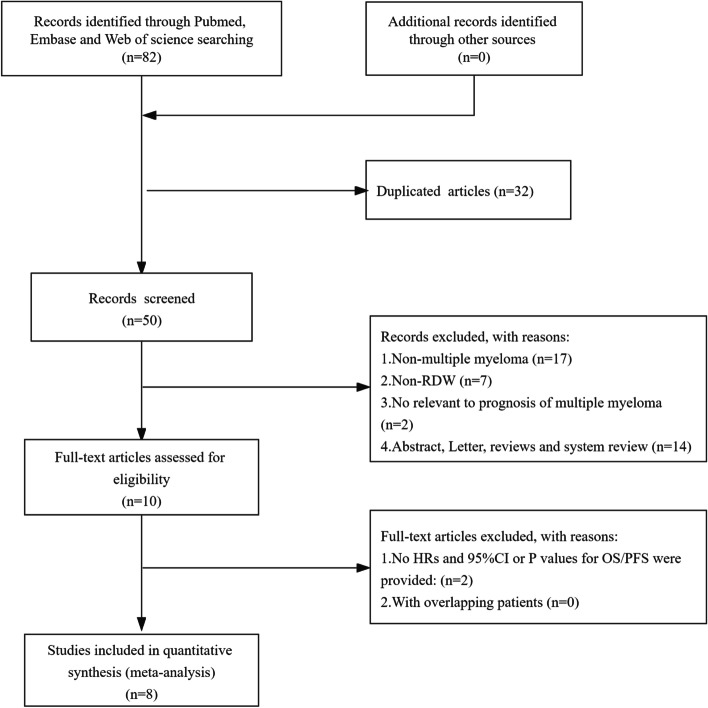
Flow chart of the included studies

The initial search strategies retrieved a total of 82 articles. After meticulous inspection of the articles, 8 articles published between 2014 and 2020 were finally enrolled in our meta-analysis [20–27]. The processes of study selection were summarized in the flow diagram (Fig. 1). One of the articles was divided into two independent samples for retrospective analysis based on whether patients were treated with bortezomib or not. Therefore, we included 8 articles involving 9 studies and 1165 patients. One study used RDW-SD for RDW, and others used RDW-CV. Among them, participants in seven studies were Chinese, and one was Korean. 8 articles directly reported HRs and 95% CIs calculated by multifactorial analysis. All included studies involved all disease stages. The characteristics of the included studies were shown in Table 1.

**Table 1:** Main characteristics of all the studies included in the meta-analysis

Study	Year	Country	Sample size(M/F)	Age(years)	Follow-up (months)	ISS stage (n)	Cut-off	Outcome	HR	NOS score
Lee H [20]	2014	Korea	146(91/55)	61(32–83)	47(3–104)	I/II/III 60/49/35	14.50	PFS	Reported(M)	8
Wang J [22]	2017	China	196(110/86)	65(33–82)	33.5(1–120)	I/II/III 6/69/71	18.05	OS	Reported(M)	8
Meng S [21]	2017	China	166 (88/78)	62(34–93)	17.79(0.63–62.83)	I/II/III 21/29/116	14.00	OS/PFS	Reported(M)	8
Zhou D [24]	2018	China	162(87/75)	61(40–87)	NR	I/II/III 35/67/60	14.00	OS/PFS	Reported(M)	7
Ma Y [23]	2018	China	78(47/31)	60.7(43–81)	42.6(2–136)	I-II/III 51/27	15.50	OS/PFS	Reported(M)	8
Sun C [26]	2019	China	99(52/47)	65(33–82)	NR	I-II/III 52/47	17.95	OS/PFS	Reported(M)	7
Liu S [25]	2019	China	175(95/80)	61	33.63(2.17–79.33)	I/II/III 23/44/108	14.00	OS	Reported(M)	7
Li DY [27]	2020	China	143(88/55)	63(35–83)	NR	I/II/III 30/43/70	47.9a	OS	Reported(M)	7

*OS* overall survival, *PFS* progression-free survival, *HR* hazard ratio, “*M*” means the HR come from multivariate analysis, *NR* not reported *NOS* Newcastle–Ottawa Quality Assessment Scale

^a^RDW was present as RDW-SD

### The prognostic value of RDW

**Fig. 2:**
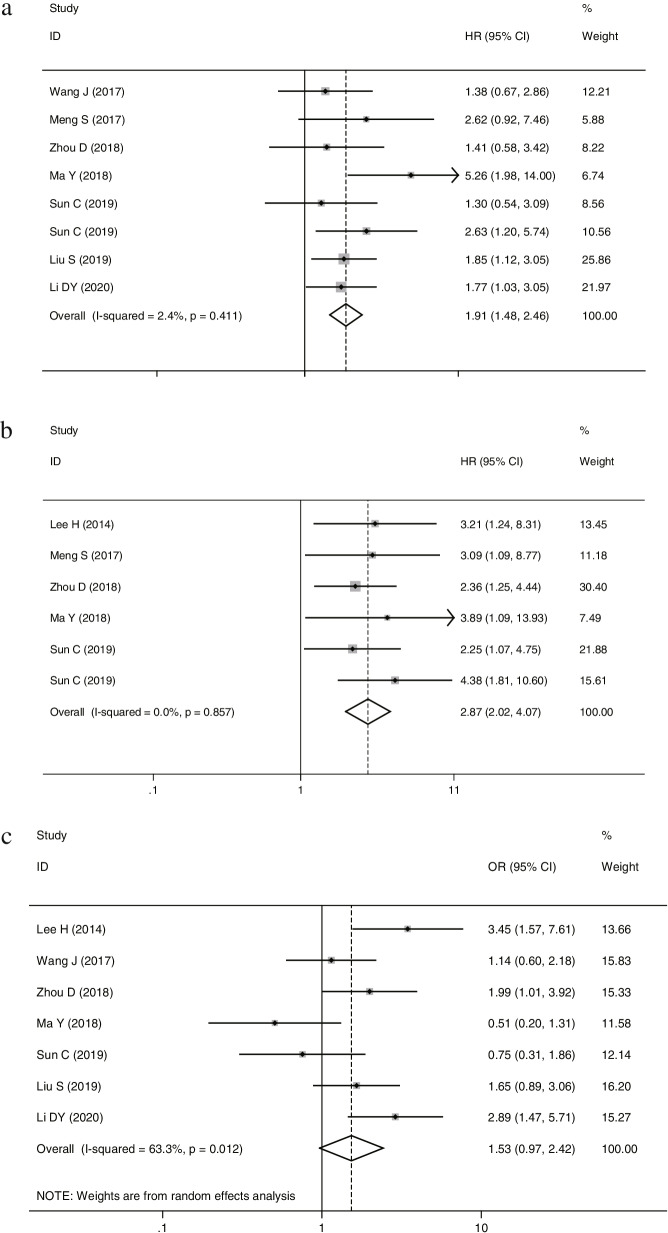
Forest plot for the association between RDW and OS (**a**) /PFS (**b**) / ISS stage (**c**) of patients with MM

8 studies reported the correlations between RDW and OS. As shown in Fig. 2a, the combined results of the eight studies suggested that elevated RDW was associated with worse OS (HR = 1.91, 95%CI: 1.48–2.46) in MM with no heterogeneity (*I*^2^ = 2.4%, *P*_h_ = 0.411) 0.5 studies reported the correlations of RDW and PFS. A fixed-effects model (*I*^2^ = 0.0%, *P*_h_ = 0.857) was also used for studies evaluating PFS. Our results revealed that elevated RDW predicted a poor PFS with a combined HR of 2.87 (95% CI: 2.02–4.07; Fig. 2b).

Then the relationship between RDW and clinicopathological parameters was analyzed using a random utility model. 7 out of 8 studies provided data on RDW and ISS staging. The results showed that RDW levels were unrelated to ISS staging (ISS III VS ISS I-II: OR:1.53; 95%CI:0.97–2.42). However, there was significant heterogeneity between studies (*I*^2^ = 63.3%, *P*_h_ = 0.012; Fig. 2c).

To analyze the source of heterogeneity, we performed a subgroup analysis of these seven studies, including sample size (< 100 vs. ≥ 100), cut-off values (< 15 vs. ≥ 15), and detection parameters (RDW-CV vs. RDW-SD). The results suggest that the sample size may be a source of heterogeneity (*P* = 0.002, Table 2). A combination of five studies with sample sizes greater than 100 suggested that elevated RDW was associated with advanced ISS staging (HR = 2.00, 95%CI: 1.37–2.92) with a small heterogeneity (*I*^2^ = 35.9%, *P*_h_ = 0.182).

**Table 2:** Subgroup analysis for ISS in MM patients with high RDW

Analysis	N	Reference	Fixed-effect model		Random-effect model		Heterogeneity		*P* value Between group heterogeneity
			OR (95%CI)	P	OR (95%CI)	P	I2	Ph	
ISS	7	[20, 22–27]	1.61(1.22,2.11)	0.001	1.53(0.97–2.42)	0.068	63.3	0.012	
Subgroup1: Sample size									0.002
≥ 100	5	[20, 22, 24, 25, 27]	1.97(1.45–2.66)	0.000	2.00(1.37–2.92)	0.000	35.9	0.182	
< 100	2	[23, 26]	0.62(0.32–1.20)	0.157	0.62(0.32,1.20)	0.157	0.0	0.551	
Subgroup2: Cut-off									0.058
≥ 15	4	[22, 23, 26, 27]	1.25 (0.85–1.82)	0.256	1.12 (0.54–2.32)	0.771	71.7	0.014	
< 15	3	[20, 24, 25]	2.12 (1.43–3.14)	0.000	2.12 (1.41–3.20)	0.000	6.2	0.345	
Subgroup3: Parameter									0.064
RDW-CV	6	[20, 22–26]	1.44(1.06–1.94)	0.018	1.37(0.84–2.23)	0.210	61.3	0.024	
RDW-SD	1	[27]	2.89(1.47–5.71)	0.002	2.89(1.47–5.71)	0.002	-	-	

*N* number of studies, *OR* odds ratio, *95% CI* 95% confidence interval, *Ph* P values of Q test for heterogeneity test

### Sensitivity analysis and publication bias

**Fig. 3:**
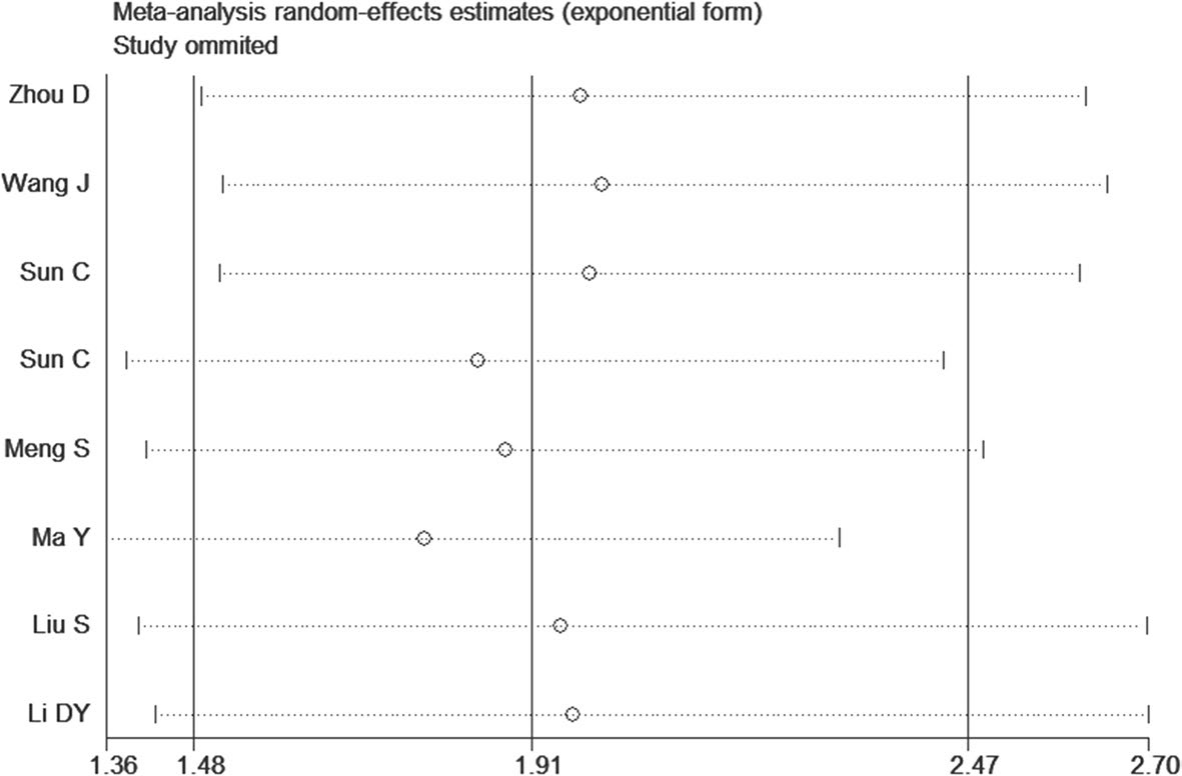
Sensitivity analysis of the association between RDW and overall survival of MM

Sensitivity analyses on RDW were performed, removing one study at a time to reveal the effect of individual datasets on combined HRs. The results showed that our findings were stable, and no study significantly affected the results (Fig. 3).

**Fig. 4:**
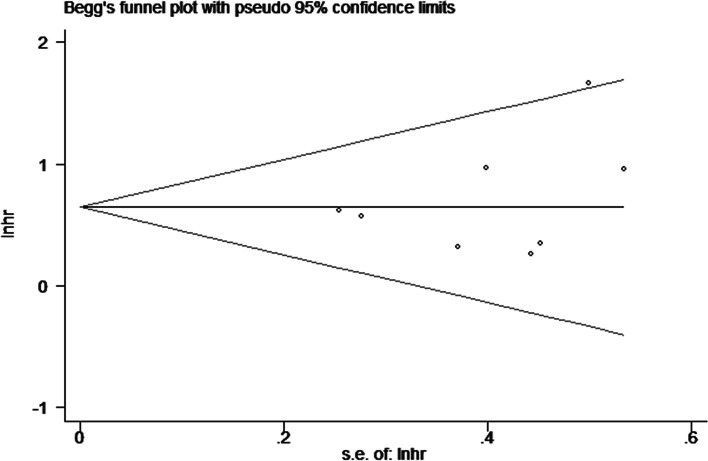
Begg’s funnel plots for detecting publication bias of the association between RDW and overall survival of MM

In order to evaluate publication bias, we performed Begg’s funnel plot and Egger’s linear regression test. There was no publication bias in the included studies from these tests (Begg’s Test: Pr >|z|= 0.260; Egger’s test: *P* >|t|= 0.116). Begg’s funnel plot was shown in Fig. 4.

## Discussion

MM is an incurable malignancy of the hematopoietic system. It is characterized by the intense proliferation of malignant plasma cells, leading to bone destruction, suppression of bone marrow function, and renal failure [28]. Increasing evidence demonstrates that chronic inflammation plays an important role in MM tumor growth and disease progression [10, 29, 30]. RDW is a parameter automatically measured in the complete blood count (CBC). RDW is an indicator of heterogeneity in the volume of peripheral blood erythrocytes [31]. RDW has recently also been the marker associated with inflammation [32]. It is considered a powerful prognostic marker in various diseases and tumors [33–35]. There remains a controversy, however, about the prognostic value of RDW in MM.

Our meta-analysis of 8 articles showed that elevated RDW predicted poor OS/PFS in MM patients (OS: HR = 1.91, 95%CI: 1.48–2.46; PFS: HR = 2.87, 95% CI: 2.02–4.07). There was no significant correlation between RDW and ISS staging. Due to the significant heterogeneity of the results, we performed a subgroup analysis. It is suggested that the heterogeneity of the included studies may derive from the sample size. Studies with sample sizes greater than 100 suggested that elevated RDW was strongly associated with advanced ISS staging. Therefore, it is reasonable to speculate that our overall study on the relationship between RDW and ISS may have a false-negative result.

The possible mechanisms underlying the correlation between elevated RDW and poor prognosis in MM are oxidative stress, chronic inflammation, cellular senescence, and poor nutritional status [36–39]. All these factors can reduce erythrocyte survival, leading to a higher mixing of circulating erythrocyte volumes [40]. RDW is an indicator of heterogeneity in the volume of peripheral blood erythrocytes [31]. Consequently, RDW is also evaluated.

Multiple myeloma cells have higher levels of intracellular reactive oxygen species (ROS) and lower levels of antioxidant molecules than normal cells due to increased oncogene activation and/or metabolic activity (e.g., high immunoglobulin synthesis rate) [41]. High levels of ROS have been shown to promote proliferation and enhance motility and invasion [42]. Decreased activity of the antioxidant molecules ARE and PON1 are considered to be biomarkers of poor prognosis in multiple myeloma patients as markers of poor prognosis [43]. Chronic persistent inflammation and inflammatory factors (e.g., IL-6) play an important role in MM [44]. Increasing evidence showed that cellular senescence and senescence-associated secretory phenotypes (SASP) establish a tumor environment that supports tumor cell proliferation and survival and promotes the development of multiple myeloma [45]. It has been established that poor nutritional status is a strong indicator of poor prognosis in MM [46, 47]. In conclusion, high RDW is a strong indicator reflecting poor prognosis in MM patients.

There are several limitations in this study that need to be clarified. The main limitation of our study was the small number of eligible studies in terms of establishing prognostic value. Second, these studies were retrospective and more susceptible to some bias. Furthermore, the power of both Egger's test and Begg's test was relatively lower when the number of studies included in the meta-analysis was less than 10. Therefore, publication bias may not be detected by Egger's test and Begg's test. It is hoped that more rationally designed, high-quality, multi-center studies will be conducted in the future to enrich our results further.

## Conclusion

In summary, this meta-analysis demonstrated that elevated RDW might be a negative prognostic factor for patients with MM.

## Data Availability

All data analyzed during this study are included in this published article.

## Abbreviations

MM: Multiple myeloma
RDW: Red cell distribution width
NOS: The Newcastle–Ottawa Scale
95%CI: 95% Confidence interval
HR: Hazard ratio
OR: Odds ratio
OS: Overall survival
PFS: Progress free survival
ISS: International Staging System
